# A qualitative study on the experiences and perspectives of public sector patients in Cape Town in managing the workload of demands of HIV and type 2 diabetes multimorbidity

**DOI:** 10.1371/journal.pone.0194191

**Published:** 2018-03-14

**Authors:** Rangarirai Matima, Katherine Murphy, Naomi S. Levitt, Rhonda BeLue, Tolu Oni

**Affiliations:** 1 School of Public Health and Family Medicine, Faculty of Health Sciences, University of Cape Town, Cape Town, South Africa; 2 Chronic Disease Initiative for Africa, Division of Diabetes and Endocrinology, Department of Medicine, Groote Schuur Hospital, Cape Town, South Africa; 3 Department of Health Policy and Administration, Pennsylvania State University, University Park, Pennsylvania, United States of America; University of North Carolina at Chapel Hill, UNITED STATES

## Abstract

**Background:**

Current South African health policy for chronic disease management proposes integration of chronic services for better outcomes for chronic conditions; that is based on the Integrated Chronic Disease Model (ICDM). However, scant data exist on how patients with chronic multimorbidities currently experience the (re)-organisation of health services and what their perceived needs are in order to enhance the management of their conditions.

**Methods:**

A qualitative study was conducted in a community health centre treating both HIV and diabetes patients in Cape Town. The study was grounded in the Shippee's Cumulative Complexity Model (CCM) and explored “patient workload” and “patient capacity” to manage chronic conditions. Individual interviews were conducted with 10 adult patient-participants with HIV and type two diabetes (T2D) multimorbidity and 6 healthcare workers who provided health services to these patient-participants.

**Results:**

Patient-participants in this study experienced clinic-related workload such as: two separate clinics for HIV and T2D and perceived and experienced power mismatch between patients and healthcare workers. Self-care related workloads were largely around nutritional requirements, pill burden, and stigma. Burden of these demands varied in difficulty among patient-participants due to capacity factors such as: positive attitudes, optimal health literacy, social support and availability of economic resources. Strategies mentioned by participants for improved continuity of care and self-management of multi-morbidities included integration of chronic services, consolidated guidelines for healthcare workers, educational materials for patients, improved information systems and income for patients.

**Conclusion:**

Using the CCM to explore multimorbidity captured most of the themes around "patient workload" and "patient capacity”, and was thus a suitable framework to explore multimorbidity in this high HIV/T2D burden setting. Integration of chronic services and addressing social determinants of health may be the first steps towards alleviating patient burden and improving their access and utilisation of these services. Further studies are necessary to explore multimorbidity beyond the context of HIV/T2D.

## Background

The World Health Organisation’s six building blocks of a health system include: governance, information systems, finance, service delivery, human resources and; medicines and technologies [1]. The ultimate goals include being responsive to the health needs of the people, promoting fairness in distribution of health services, protection of families from catastrophic health expenditure and respecting people. In response to the need for strengthening health systems for chronic care, conceptual models have been developed such as the Innovative Care for Chronic Conditions (ICCC) model [2], the Integrated Chronic Disease Management (ICDM) [3] and the Cumulative Complexity Model (CCM) [4].

The Innovative Care for Chronic Conditions (ICCC) model was conceptualised by WHO in 2002, which emphasises the importance of offering quality healthcare services, the integration of chronic conditions services and adaptability to changes in burden of disease [2]. In line with the ICCC model, literature on low to middle income countries (LMICs) show that there have been repeated calls for a shift from vertical disease programs to joint management of chronic conditions in order to reduce the burden of care on both the individual patient and the health system [5–10] with few empirical studies done in sub-Saharan Africa to assess the feasibility of such innovations [11, 12]

In South Africa (SA), the ICCC was adapted through formulation of the Integrated Chronic Disease Management (ICDM) framework between 2011 and 2013. The ICDM argues that optimal clinical outcomes for people living with single or multi-morbid conditions can be achieved through primary healthcare (PHC) re-organisation involving improved clinical management support, clinical practice guidelines for integrated care and the use of community healthcare workers (CHWs) to assist patients with self-management [3]. In addition, it is a refinement of the national strategic plans for communicable disease (CDs) and non-communicable diseases (NCDs) [13] [14] in that the convergence of CDs and NCDs is acknowledged in the recommended organisation and management of chronic health services. To our knowledge, no studies have been done to examine the ICDM.

While these two frameworks propose strategies to integrate management of multiple chronic conditions, neither the ICCC nor the ICDM detail the complexity of patients’ lived experiences as they interact with the healthcare system. The Cumulative Complexity Model (CCM) [4] was developed as a "patient-centered framework to guide improvements in the analysis and evaluation of patient-related complexity in the management of chronic conditions, and to promote innovative healthcare delivery for these patients.” The model has four major interrelated elements; patient workload; patient capacity; access, utilisation and self-care; and health outcomes. These become intertwined by the feedback loops of burden of illness and burden of treatment.

Central to this model, is the acknowledgement of the workload of demands related to chronic disease management (“patient workload”) that is associated with living with co-morbid conditions. Examples include daily tasks and responsibilities related to self-care, job and family. A patient’s capacity to meet this workload (“patient capacity”) is determined by capacitating factors such as their physical or mental functioning, socioeconomic resources, social support, level of literacy and attitudes or beliefs. The interaction between workload and capacity affects a patient’s access and utilisation of healthcare services and the ability for self-care; which ultimately influences health outcomes. Health outcomes are also influenced by the feedback loops of the burden of treatment and burden of illness that contribute to the delicate balance between workload and capacity

HIV and type 2 diabetes (T2D) multimorbidity is an example of CD/NCD convergence in the South African health system [12] [15] [16]. The need to incorporate feedback from patients on their interaction with the health service and capacity to understand and absorb demands related to self-management of their chronic multi-morbidities in their home environment were conclusions drawn from a multimorbidity study in Zambia [17]. However these aspects remain underreported in South Africa. The concepts of the CCM such as the burden of treatment [18] and capacity [11] have only been explored in high income settings. Herein we describe a study in which this model was applied to the examination of individual’s experiences of HIV/T2D multimorbidity in the SA context.

## Methods

Ethics approval for the study was granted by the Western Cape Department of Health and the Human Research Ethics Committee of the University of Cape Town (HREC Ref: 314/2015). This was an exploratory, qualitative study involving face-to-face, in-depth interviews with individual patient-participants and healthcare workers. Phenomenology underpinned the study as the interviews drew subjective lived experiences and perspectives of a sample of patients and healthcare worker participants in managing the workload of demands of HIV /T2D multimorbidity; and how this influenced the patient participants’ capacity for effective self-management. The lines of investigation followed the conceptual framework of the CCM [4]. The data will be used to develop a more detailed funding proposal and protocol for further research.

### Setting and study population

The study was conducted in Khayelitsha, a peri-urban, largely informal township of predominantly black, Xhosa speaking South Africans in Cape Town. Although 62% of the labour force is employed, 74% of household income is less than R3 200 per month. Within this community, 45% of households live in formal houses and 62% of households have access to piped water in their yard [19]. For this study, all participants were drawn from two, public sector clinics situated at Site B PHC facility in Khayelitsha, namely: Ubuntu Clinic and the Site B community health clinic. Ubuntu clinic provides HIV and TB services; whilst Site B community health clinic provides primary health care for all other diseases, including T2D.

### Sampling

Convenience sampling was used for both patient and healthcare worker participants because the researcher had to rely on available participants at the health facility. Purposive sampling was also used as the researcher recruited participants from the health facility based on the eligibility criteria. Eligibility criteria for inclusion into the study included: patients with both HIV and T2D multimorbidity; having initiated antiretroviral therapy (ART) and also be on treatment for T2D; and between 35 and 65 years (those below 35 years have a higher chance of having type 1 diabetes and those above 65 are the least affected in this setting). Healthcare workers had to be working with adult patients with chronic multimorbidities. Additionally, all participants had to be capable and willing to: provide verbal and written informed consent; and communicate in simple English. Due to the exploratory nature of the study and limited budget, the total number of participants enrolled for the study between July and August 2015 were ten patient-participants and six healthcare workers (*N* = 16). Fourteen patients were approached and ten provided consent; whilst nine healthcare workers were approached and six agreed to participate in the study. Equal numbers of males and females were recruited among patient-participants. Healthcare workers included two doctors from the general clinic, two clinical nurse practitioners (CNPs) (one from the general clinic and one from the HIV clinic) and two HIV counsellors (from HIV clinic). There were no available doctors from the HIV clinic during the period of recruitment.

### Data collection

Interviews were in-depth, semi-structured, and face to face and conducted by RM the lead author, in a private room in the clinic. They were conducted in English, audio-taped and lasted for approximately an hour. As Xhosa is the dominant language in Khayelitsha, a translator was present in each patient-participant interview to provide translation assistance if the research participant needed to ask or answer questions in the vernacular. Before interviewing study participants, we conducted 2 pilot interviews with patients that attend a diabetes clinic located at a tertiary hospital in Cape Town. Interviews were guided by two separate semi-structured questionnaires: one for patient-participants and the other for healthcare workers. The questionnaires were based on the themes of the CCM that explored the concepts of "patient workload" and "patient capacity". Patient-participants were asked what they had to do to care for their health, the challenges they faced in meeting these demands and the factors that helped them. Healthcare workers were asked how they provided care for HIV/T2D patients, the challenges experienced in this interaction and how they assisted in developing patient capacity. As interviews were once off and no transcripts were returned to participants for comments, RM made field notes after every interview to identify areas that may need further probing with subsequent interviews. Interview transcriptions were verbatim and in English. Patient-participants’ names were replaced by numbers in the transcriptions to ensure confidentiality. At the end of each interview, a food voucher to the value of R100 was given to patient-participants as a token of appreciation.

### Data analysis

NVvivo computer software was used to manage the data. Data analysis was primarily done by RM with assistance from a qualitative research expert (KM). Further discussions with the qualitative research expert enabled RM to be reflexive of assumptions and biases that may have influenced the research process. Thematic content analysis was applied to the transcripts [20] [21]. This involves the researcher becoming familiar with the data through reading the data, reflecting, coding and refining codes. Deductive codes from the CCM were used; together with inductive codes derived from the data. A codebook was developed to harmonise data derived from individual interviews [22]. Data from participants were then described and compared. Lastly, data were extracted and explained by in relation to the existing literature.

## Results

### Demographic data

Demographic data were recorded for all participants at the beginning of each interview. Patient-participants had been on treatment for HIV for 8.2 (5.0) years and for 9.4 (6.0) for diabetes. HIV medication was a 3-in-1 combination pill that was taken orally once a day by nine of the participants; yet one patient-participant reported having one tablet in the morning and two pills in the evening. Patient-participants injected insulin (*N* = 7); and subsets took three (*n* = 2) and four (*n* = 2) extra tablets orally every day. The remaining patient-participants (*N* = 3) took one to three pills per day for T2D. Hypertension was self-reported by 60% of the patient-participants. One patient-participant was educated up to primary school level and another had a tertiary qualification; with the remaining eight educated up to secondary school level. There was an equal distribution of unemployment and employment among patient-participants with those employed working in household work and security services. Among these patient-participants, 70% lived in informal housing, which they shared with one or more family members (Table 1).

**Table 1:** Patient-participants’ demographic data (*n* = 10).

Age	*SD*
	*46*.*9 (8*.*8)*
HOUSEHOLD AND ENVIRONMENTAL VARIABLES	*n* (%)
Housing	
Type: ^♦^ Formal	3 (30%)
^•^Informal	7 (70%)
Ownership: Self	5 (50%)
Relative	5 (50%)
Water supply: On own property	6 (60%)
Communal	4 (40%)
Number of patient-participants who specified their household members	
Partner	7 (70%)
Child(ren): Minors (0-18years)	3 (30%)
Adults(>18years)	3 (30%)
Grandchildren (0-18years)	3 (30%)
Relatives (>18years)	2 (20%)
Siblings (>18 years)	1 (10%)

^♦^Formal housing is made of permanent material such as bricks

^•^Informal housing is made of temporary material such as steel sheets and plastic

Healthcare workers were educated up to tertiary level with the two doctors progressing to postgraduate level. It was also important to note that the doctors had more years of work experience in comparison to the CNPs and HIV counsellors. HIV counsellors work experience ranged from four months to three years; for CNPs, three to twenty years; and for doctors, six to thirty years.

### Interview results

Patient workload and patient capacity as conceptualised in the CCM, varied among participants; and they had different reflections on how to improve access and utilisation of healthcare services for multimorbid conditions. (Table 2). These themes were identified during the interviews and when the transcripts were being reviewed.

**Table 2:** Summary of interview findings.

Self-reported experiences by all participants on access and utilisation of HIV/T2D healthcare services and self-management	List of strategies to improve patients’ access and utilisation of HIV/T2D healthcare services and self-management
***Theme 1*: *Perceived patient workload***	
***Clinic-related workload***	
-Separate HIV and T2D clinics	-Integrated health services
-Long waiting periods and incomplete clinic visits	-Improvement in healthcare worker lunch break rotation, provision of community-based healthcare services, employment of more healthcare workers
-Inefficient computer system	-Integrated information systems
-Separate HIV/T2D clinical guidelines	-Consolidated guidelines
***Self-management workload***	
-Nutrition	-Purchasing food and fruit from vegetable vendors
-Taking medication	-Routine, personal daily reminders
-Physical activity and personal safety	-Routine
-Stigma	-Patient education
***Theme 2*: *Perceived patient capacity***	
***Positive attitudes*** -Having confidence in medical treatment -Support from family -Strong beliefs in religious or cultural practices	-None suggested
***Health literacy***	-None suggested
***Family support*** -Accompanying patients to clinic visits -Partner/spousal support	-None suggested
***Clinic-based support*** -Availability of educational materials that appeal to patients with different physical and mental functioning, age and education levels -Dietary supplementation for underweight patients -Availability of established advocacy groups -Availability of suggestion boxes and an “open door: policy” by the general clinic manager	-Healthcare worker-patient relationship building through: individual and group support groups for younger and older patients -Healthcare workers and family members to be empathic of patients’ health outcomes -Continuity of care and continual professional development of healthcare workers -Responsiveness of healthcare facility managers to patients’ comments and queries submitted in suggestion boxes on health service utilisation
-Financial challenges to meet nutritional and clinic visits expenses	-Government grants, community cooperatives

### Theme 1: Perceived patient workload

#### HIV and diabetes clinics-related workload

Patient and healthcare worker participants reported that at the time of interviews, there was no integration of care for patients with HIV/T2D multimorbidity as administrative and consultative functions for HIV/T2D clinic visits were done in two separate clinics on separate days. In primary healthcare, patients with chronic morbidities are assigned into “adherence clubs” and “non- club patients”. The adherence club system operates for patients with a chronic disease who are considered stable as their disease is optimally managed, their adherence levels are high and are on treatment for more than a year. It aims to streamline their clinic visits and improve waiting times, disease management, and adherence. Non- club patients routinely attended the clinic once a month and more frequently, depending on whether either disease was not optimally managed, if adherence levels were low, or if treatment was recently initiated.

**HIV Clinic:** Adherence club patients (ART<1year) have fixed membership, meaning each club consists of the same patients (approximately 35 per club). When a patient is lost to follow-up, the doctor or CNP assigns another patient who is not yet in an adherence club to fill the position. One “non-club” patient participant (newly diagnosed: ART>1year); together with the remaining nine adherence club patient participants visited the HIV Clinic in the morning any time after 8am. They found the average wait of a maximum of two hours for a full consultation (registration and vital checks, visit to the CNP and/or doctor; and dispensation of medication) acceptable. However, healthcare workers often did not have a holistic picture of patient data, such as previous medical test results on file as noted by one patient-participant:

*“… when they (nurses) do blood tests*, *we have to remind them that last time I was here I did a blood test*, *so can I get the results*. *Now I’m thinking it's not necessary for the nurse to ask you again*, *“When did you last take the blood test; are you willing to be informed about your results?”*

*–Participant P8*, *male*

**Diabetes Clinic:** The diabetes club system operated on an open membership basis where patients had to show up on a club day based on the manually-assigned appointment made by the doctor. This often resulted in overcrowding in the club room and an unbalanced allocation of patients for each club day:

*“There are a couple of issues with the booking system …some Mondays we'll get two hundred people and then on some Mondays we get eighty people in the club*. *And the reason this happens is because those giving out the dates to come back don't have access to the numbers that are coming back on that day.”*

*- Participant H2*, *doctor*

All healthcare workers and nine patient-participants also reported on the burden of long waiting periods during diabetes clinic visits. Patients left their homes for the diabetes clinic very early in order to queue before the clinic opened at 8am and a full consultation took six to eight hours. Sometimes, patients waited for an additional hour while the healthcare workers had their lunch break; and additional appointments were given for another day to see the doctor, counsellor, dietician or consultation for any other illness. Patient-participants described the waiting as exhausting, frustrating, a threat to the security of their jobs and a waste of their time. The situation was made worse when they would arrive at the pharmacy too late to get their medication or to be told that none was available due to a stock-out:

*“…Sometimes we don't even receive our medication on that day that you came for the clinic*. *They will give you a letter that tells you*, *you have an appointment tomorrow morning.”*

*–Participant P3*, *female*

#### Strategies to improve patients’ access and utilisation of HIV/T2D healthcare services

Patient-participants suggested comprehensive clinic visits which would address all their health concerns at one time to improve their satisfaction during clinic visits. In their view, shorter waiting periods could be achieved through improving operations in the health facility and the provision of community-based services. For instance, patient-participants proposed scheduling healthcare workers lunch breaks at the health facility in a way that allowed continual attendance throughout the day. In the community, one patient-participant suggested having mobile clinics for T2D and referrals to the PHC for complicated cases. Additionally, another patient-participant preferred CHW services such as home visits to deliver medication, adding that this would benefit not only the elderly but all patients with chronic morbidities. However, both healthcare workers and patient-participants acknowledged that a shortage of healthcare workers contributed largely to the current healthcare service provision and limited community-based services thereof; with three healthcare workers and one patient-participant suggesting that the national government urgently addresses this need:

*“And the staff is too little*. *So I know that our managers and Government are aware of that.”*

*- Participant H6*, *CNP*

The need of integrated clinical guidelines and an improved computer system that is accessible to all healthcare workers to better manage HIV/T2D were suggested:

*The only way we can integrate that is if you have a computer system*. *So if I have a computer here*, *I can access reports from Ubuntu* [HIV clinic] *so that I can see what their CD4 count is*, *what was done last time and so on at least …We got guidelines for diabetes and there are guidelines for HIV*, *but there are no integrated guidelines*. *So how do you question management of HIV and diabetes if there is no unique guideline for that*? *Or HIV*, *diabetes and MDR* [TB] *when there is no specific guidelines for it?”*

*- Participant H1*, *doctor*

#### Self-management workload

**Nutrition**. Health education provided at the two clinics was central in shaping patient-participants' knowledge for HIV/T2D nutrition; but adherence to recommendations was often challenging. Patient-participants regarded the healthy dietary recommendations given by healthcare workers at the two clinics to be the same for managing their multimorbid conditions. Patient-participants placed greater emphasis on T2D nutrition since they were more susceptible to unpleasant T2D-related symptoms if they had poor nutrition.

Except for one patient-participant, all participants reported that they shared the same food with their families and struggled to afford the recommended foods. In most cases, this situation forced patient-participants to forego the ideal diet for their chronic conditions:

*“ …your rice is not right*, *potatoes are not right*, *mealies are not right and that is the only food that we eat*. *So we can't afford that stuff.”*

*- Participant P2*, *female*

*“…sometimes she* [wife] *has to make two pots*, *because when I say to her*, *‘Please when you cook for me*, *cut the skin before you cook’ she is not diabetic* [and] *she says*, *‘The meat is much more enjoyable with the skin*.*’ So it’s a challenge in every family because being diabetic you adjust your life*, *but when you are not it’s not easy.”*

Participant P8- male

**Taking medication:** Taking medication constituted a major task for patient-participants in order for them to maintain optimum health outcomes. The most commonly reported symptoms by patient-participants were dizziness, night sweats, psychological distress; and clinically-diagnosed complications such as lipodystrophy, renal failure, and hypertension. These were largely due to disease progression, diet and the side effects of drugs and drug-drug interactions. All patient-participants struggled at the time of diagnosis with the pill burden, but this diminished over time and they eventually did not feel overburdened to manage their health

“I found it difficult at the time that I'd just been diagnosed with diabetes…to inject myself and I have to take a certain amount of pills… but now I became to use to it and now I'm just living life”

*-Participant P1*, *female*

**Physical activity and personal safety:** Adherence to physical activity recommendations and ensuring personal safety did not appear to be burdensome to patient-participants who responded to this theme.

**Stigma:** Four patient-participants experienced HIV-related stigma within the family and community at point of diagnosis:

*“He* [spouse] *didn’t even want to see me* [and] *didn’t even want wind coming from me towards him*. *He had me stay at the back of the house… Even now I still stay in a shack*, *but now it's much better*, *much happier*. *It was a hard time that time”*

*- female*, *HIV-related stigma; HIV*: *14 years*, *T2D*:*8 years*

Although patient-participants reported that family members and the community were more accepting of HIV than in the past, one patient-participant experienced T2D-related stigma and did not disclose his HIV status to his relatives as he feared further stigmatisation:

*“I didn’t tell anyone*. *I don’t want to tell them*, *because sometimes even my family is not alright*. *If you clash*, *they say this guy has it* [HIV]…*but they know I’ve got diabetes and the way they talk hurts*. [They say] “W*here are you getting the diabetes when you are small*?*” I tell them I did not call for the sickness*.*”*

*- male*, *HIV*: *9 years; T2D*: *5 years*

Another patient-participant also feared HIV/T2D stigma and had not disclosed his HIV/T2D status to people outside the immediate family:

*“When one receives such information*, *you only tell your family*. *That’s the basic*. *After that*, *because going and telling everyone that you are sick*, *that’s not going to do anything for you.”*

*- male*, *HIV*: *6 years; T2D*: *15 years*

Amongst healthcare workers, four and one mentioned that patients were still experiencing HIV and T2D related stigma, respectively.

#### Strategies to improve self-care

**Nutrition:** Patients were advised by CNPs and doctors to follow a strict diet that included: drinking water instead of fizzy drinks, eating less carbohydrates, using less salt, substituting sugar with diabetes sweeteners, having less fatty meat and eating more fruit and vegetables. Patient-participants also mentioned the importance of ideal portion size and frequency of meals on a given day:

*“I must eat five times a day*. *Not like three times a day and making six slices*. *I must make like small portions*. *But I must make sure my starch is less and less*, *because according to her that is causing my weight to rise.”*

-*Participant P8*, *male*

Healthcare workers from both clinics were conscious that many patients could not decide on food options due to family dynamics such as who the patient lives with and finances. A doctor pointed out the importance of educating family members so that the whole family knows of the implications on health outcomes for a family member who has T2D. Additionally, if a family could not afford buying the required foods, recommendations were given to patients on affordable options and explaining that often the problem was the way food was prepared:

*“I tell them that you can get these things at the umababela* [the street vendor] *because you get them cheap there; spinach*, *cabbage*. *It is also how to cook them at home*, *because they like to fry*, *they like the beef stock*, *they like the Aromat… you eat the cabbage as green as it is …”*

*–Participant H5*, *CNP*

**Taking medication:** Apart from one patient-participant who was uncomfortable taking insulin, further actions mentioned which indicated that patient-participants had integrated taking medication into their daily routine included setting alarms to remind them to take their medication and taking medication just before meals.

**Physical activity and personal safety:** Three patient-participants mentioned that they had developed a daily routine of walking in order to manage T2D. Two other patient-participants said that they danced or jogged to lose weight:

*“There was a time that my weight used to be hundred and ten* [kilograms], *but now*, *ever since I started exercising it's gone down to ninety-four*, *so exercising helps*. *Even if you don't have to walk around the place—just being at home and dancing is exercise.”*

*- Participant P3*, *female*

Patient-participants practiced personal safety for both HIV and T2D. For HIV, patient-participants spoke about safer sexual practices and the need to protect open wounds whilst for T2D, one patient-participant mentioned the need for proper foot care, which included washing and drying feet properly; and also wearing the right shoes "to be safe".

**Stigma:** Two healthcare workers suggested further patient education to be helpful in diminishing HIV/T2D stigma:

*“Some of them are saying*, *“I don’t think I will disclose now; I can’t tell my boyfriend otherwise we will end up separated” So it’s even then I try to convince the patient that*, *“You have to tell*, *you have to be strong…it’s very important to do that*. *It must not be you only one who knows about it*. *But I don’t have to push you*. *Do this in your own time*.*””*

- Participant H3- HIV counsellor

*“I think one other thing is*, *you know people don't want to disclose*. *Let's say I'm looking culturally*, *I'm looking at a man*, *who is diagnosed with diabetes …I had a newly diagnosed*, *family man and he asked me*, *“Sister*, *what is going to happen to me now*. *I know there is the loss of libido; because I have heard from so and so that he has a problem and now is that going to happen to me*?*” So I said people are different*. *It is not always*, *and it is not everyone that has that problem ”*

*–Participant H5*, *CNP*

### Theme 2: Perceived patient capacity

#### Capacitating factors for patients

**Positive attitudes:** All patient-participants believed positive attitudes were essential to accepting and coping with living with HIV/T2D. Patient-participants reported that having confidence in medical treatment; support from family and strong beliefs in religious or cultural practices all helped them psychologically to come to terms with their morbidities. The positive diagnoses of HIV and T2D were difficult at first, as it affected them physically and mentally, but all of them felt that they had adjusted well over time:

*“That time that I just found out this condition*, *that I’ve got these diseases…I felt very*, *very low at that time*. *I felt like I couldn't accept the way that I had to live*, *but as time went by*, *I accepted those things and that's what I am now.”*

*- Participant P1*, *female*

**Health literacy:** Health literacy for HIV/T2D management varied by the patient-participant’s physical and mental functioning, age and education levels. For instance, one patient-participant argued that it was their responsibility to make sure they were well informed; whereas another had little understanding of HIV:

*“It also depends on your curiousness*. *If you are not curious enough then the information is going to be far away from you*. *You have to be inquisitive and ask how HIV works.”*

*- Participant P8*, *male*

*HIV I really don’t understand*. *The clinic guys wouldn’t be able to tell me about HIV*. *I was told its just dirty blood*, *that my blood is dirty… I was given a huge book to read and there were things like signs*. *I was just told there is no cure; that this is something I have to accept now*. *There’s only medication that can just keep it*.”

*–Female*, *educated up to primary school level*

A healthcare worker also indicated the challenges of mapping out a self-management plan for psychiatry patients:

*“*[A psychiatry patient] *has got about ten medications to take every day*. *So now she is coming with a blood pressure of over two hundred* [mg/dl] *and her blood sugar is uncontrolled*. *So I ask her how many ARVs are you taking*. *She says three*. *So I don't know if it is the combination tablet and the others are vitamins or are it three different drugs*, *I don't know.”*

*- Participant H1*, *doctor*

Healthcare workers reported using health education methods on HIV/T2D management to patients such as oral presentations, visual aids and written material at an individual level during consultation with CNPs or doctors; and group level during club visits for either health condition.

*“So they have that pamphlet which they take home*. *We always tell them*, *“Put it on your fridge*, *in your face where you'll be able to see; so that something will pinch* [remind] *you if you're still doing what you're not supposed to do.”*

*-Participant H5*, *CNP*

Although one patient-participant maintained that no health education materials were available in both clinics, the remaining participants recalled seeing posters or pamphlets on both HIV and diabetes clinics:

*“They mostly work with the posters*, *whereby they show us this*, *the different foods.”*

*–Participant P4*, *male*

*“…they have the different foods that we eat and the ones that are not right for us all over the club*. *I understand*, *because there are pictures and writing also.”*

*–Participant P5*, *male*

**Family support:** One healthcare worker indicated that family members were supportive in accompanying patients on their clinic visits. To patient-participants, family support was important and where it lacked in some areas or totally unavailable, it caused distress in managing HIV/T2D:

*“ …I get full support from my children*. *There’s a unity of strength* [in my family] *“… but it's very difficult when you’re alone*. *If only you had your better half then it would be easier*. *He would take care of some of the things and you would relax*. *So it's not easy being alone*. *To handle everything*, *to sort everything.”*

*- Participant P6*, *female*

“…my sugar goes up because my wife always shouts at me.”

*–Participant P7*, *male*

**Clinic-based support:** Three patient-participants and two healthcare workers viewed clinical support as adequate for HIV/T2D management; but four patient-participants and one healthcare worker had contrary experiences. Participants in favour of this view appreciated the health education services mentioned earlier; and people living with HIV (PLWHIV) with very low body-mass index (BMI) were referred to the dietician for a food programme for dietary supplementation. Additionally, external advocacy groups stationed permanently at the HIV clinic such as Treatment Action Campaign (TAC) championed the rights for all PLWHIV and Mothers2Mothers had empowering programmes for mothers living with HIV.

Two of the four patients elaborated that the bread and soup services during clinic visits for T2D were unreliable and not sustained. One healthcare worker noted that referral of patients to the psychiatrist, dietician or social worker was difficult as these services were under-resourced, infrequent and in high demand.

Additionally, the health services at both clinics had taken initiatives to be more responsive to patient complaints and queries. Suggestion boxes were available but one patient complained that the suggestion boxes were ineffective due to lack of feedback. The facility manager at the diabetes clinic was also open to meeting patients:

*“We have the manager’s office that is open to everyone*. *Should the person be not satisfied*, *he or she needs something to be done now*, *we send them to the manager's office”*

*–Participant H5*, *CNP*

**Finances:** Some patient-participants were unable to meet their daily needs for HIV/T2D management because of financial constraints. Apart from nutritional challenges associated with low household income, all patient-participants incurred double the travelling costs from home or work to the clinic for their separate HIV/T2D clinic visits. Depending on affordability, they travelled by taxi (40%), train (10%), private car (10%) and unreported (20%). Three patient-participants were in full time employment and were financially self-sufficient. However, two relied on a disability and old age grant, respectively; with the rest dependent on family members for survival.

#### Strategies to enhance patient capacity

To improve HIV/T2D management, participants shared different ideas on enhancing clinic-based support and patient income.

**Clinic-based support:** However, clinical support was inadequate as other patient-participants articulated a need for: 1) further information and counselling about the implications of having children if one was HIV positive (*n* = 1); 2) separate T2D support groups for younger and older patients (*n* = 1); and 3) greater empathy from healthcare workers for their struggles in managing their blood glucose (*n* = 2).

For patients to be more open about their HIV/T2D management and also promote continuity of care, doctors suggested each patient be assigned to one or two doctors or CNPs for all clinical consultations. As the doctors highlighted, this would entail further training such as mentoring junior doctors to prescribe medication rationally to patients.

**Finances:** A doctor called for the revision of the eligibility criteria for the disability grant as qualification was easier for people who had physical disabilities than those with non-physical disability. Two other healthcare workers and advocacy groups supported the idea as they viewed provision of disability grants to all patients with chronic conditions would help patients adhere to a healthy diet:

*“TAC wrote a letter to our government and we signed in support …The proposal was for everyone who is unemployed and HIV positive*, *to get a grant—maybe a payment of a R1 000 from Government every month.”*

*- Participant H6*, *CNP*.

However, one healthcare worker disagreed:

*“I don’t think it’s necessary to provide them with any extra funding or anything like that to eat healthier*, *because there is food*: *fruit and vegetables are available and cutting sugar- that doesn’t cost a lot of money.”*

*-Participant H2*, *doctor*.

Two patient-participants pointed out that food gardens could be a means of food security, yet two other patient-participants perceived this as impossible due to inadequate water supplies in their community, lack of space and other inputs required in producing food.

*“It's because at the place I’m staying* [informal settlement] *there are not a lot of yards so that we can have vegetable planting.”*

*–Participant P7*, *male*

## Discussion

As separate clinics exist for HIV and T2D care, the study confirms that the envisioned integration of care at a primary provincial care level for CDs and NCDs according to the new policy objectives of national government is currently under-developed in the public sector primary healthcare services in Khayelitsha—an indication of the many difficulties involved in re-organising health services. To explore patient and healthcare worker experiences of chronic disease management in the context of multimorbidity, this study utilised the CCM [4]. Issues of experienced patient workload, factors that influence patients’ capacity to manage chronic multiple conditions, and the roles these experiences play in the access and utilisation of health services; as well as patients’ ability to self-manage their conditions, were interrogated.

### Patient workload

Patient workload were reported as either being clinic-related or related to self-care. With respect to clinic-related workload, patient-participants still experienced disparities in health services for HIV and T2D. For example long waiting periods and healthcare worker shortages were the most frustrating issues raised by participants at the diabetes clinic, unlike the experiences of patients at the HIV clinic. Indeed these factors have been long-standing problems for users of PHCs since the late 1990s in South Africa [23]. In the context of HIV health services, advancements have been made through the ARV adherence club system; which avoids overcrowding and reduces waiting times at the health facility [24] and this was reinforced in our study. In the short term, adopting a similar adherence club system for diabetes care and progressively a single adherence clinic for T2D, HIV and other highly prevalent chronic morbidities in this setting is needed in order to improve patient satisfaction of using chronic diseases healthcare services.

Leveraging ancillary services in the community is helpful to lessen pressure on the PHC such as CHWs supervising uptake of treatment and providing palliative care especially for HIV and TB in Cape Town [25]. However, challenges such as poorly developed systems between community based service providers and the formal public healthcare services [26]; and unsustainable financing for integrated chronic diseases health services [17] remain endemic to sub-Saharan Africa in efforts to roll out community-based services for both chronic CDs and NCDs. As intersectoral action between state and non-state organisations largely contributed to the success for single disease management such as HIV programmes across LMICs [27] [28] [29], adopting such an approach for integrated management of chronic diseases is likely required to improve access and utilisation of healthcare services for people living with multimorbidities.

Self-management workloads experienced in our study such as stigma and adherence to prescribed T2D diets appeared to be greater tasks for patient-participants than taking medication, physical activity and personal safety. Patient-participants experienced more HIV-related stigma than T2D and findings elsewhere in South Africa show HIV disclosure to be received with positive reactions by other family members who are not sexual partners resulting in optimal HIV management by both the patient and the respective supportive family members [30]. Focusing on nutrition, culture and socioeconomic status largely shaped patient-participants’ eating patterns; especially for T2D. Our findings were similar to results from a study in Senegal where culture was associated with patients failing to adhere to an ideal diabetic diet since having a different diet is sometimes unaffordable, makes patients feel isolated and having reduced food portions feels like abandoning the common family practice [31] [32]. Furthermore, from the South African 2015 general household survey, the percentage of households still experiencing limited access to food remains high (22.6%) [33] with food security studies on urban areas attributing access to food as the major driver of food insecurity [34] and most of the unhealthiest food options that promote diet-related NCDs [35] [36].

The challenges in managing stigma and nutrition highlight the importance of medium and longer term and sustainable upstream approaches that address these social determinants of health for patients with multimorbidities to improve health outcomes. For instance, although HIV/T2D education is established in the current healthcare provision; health literacy for multimorbid disease management and reducing stigma within communities and among patients can leverage social participation through redesigning education modalities that cater for different education levels. National governments in both high income and LMICs have also adopted public health focused fiscal measures, nutrient, food, and diet standards [37] [38] [39] that make it possible for people to access healthier nutrition to prevent and reduce diet-associated complications of CDs. However, further collaboration between state and non-state actors in LMICs is needed to ensure food security among households that have patients living with multi-morbidities and low socioeconomic status. Such steps may include acknowledgement of the informal sector such as street food as a source for promoting healthy food security [39] [40] and training on food safety strategies as done in Ghana [41] and India [42]; and increasing fair and decent employment opportunities [43] [44]

### Patient capacity

Participants’ suggestions on how to improve patients’ ability to self-manage HIV/T2D multimorbidity were largely around health literacy, family and clinic based support; and the availability of finances. Importantly, the feedback loops illustrated in the CCM between patient workload and patient capacity were strongly visible between the relationship between stigma and nutrition; and health education, family support and finances. Hence, health literacy, family support and socioeconomic factors as capacitating factors to manage multimorbidity are discussed earlier. With reference to clinic-based support, our study indicated the importance of relationship building between patients and healthcare workers to improve HIV/T2D management. Trust, between healthcare workers and patients, was identified as an important requirement for effective clinic-based support. In a systematic review, an established long-term relationship with a single individual or team of health care professionals was associated with improved quality of care for patients with chronic multi-morbidities [45]. However, a significant challenge to achieving this in LMICs is the chronic shortage of healthcare workers within the PHC system that needs to be addressed first.

Another key issue identified was the patient: health care worker power divide. For example, patient-participants perceived they did not have authority to question healthcare workers for leaving them unattended in the waiting areas when healthcare workers took their lunch breaks. While regulations stipulate the need of a one hour meal interval after working continuously for five hours [46] a functional system of rotation so that there is a balance between healthcare workers on lunch break and those attending to patients was seen to be ideal by patient-participants. Healthcare workers perceived patients’ posting written compliments and queries on healthcare service provision into suggestion boxes to be a mechanism of demonstrating patient power but patient-participants deemed this platform unsuccessful, as they did not see changes or receive feedback. To increase patient satisfaction and improve quality of patient care, the healthcare worker-patient relationship has to be reciprocal such that patients have client power towards health providers and healthcare workers have an obligation to provide service to the people [47].

## Limitations

The paucity of evidence in CD/NCD convergence and lack of empirical work examining the CCM limited comparison of our findings to other studies. Although our findings were internally valid, a further limitation is the small number of participants and inclusion of only one setting, limiting our ability to achieve the data saturation required to fully explore all the different dimensions of the CCM. This may limit the generalisability of study findings to other settings. It is possible that more interviews could have yielded further issues of relevance, a greater breadth of information and deeper insight on this topic. Lastly, as optimal HIV/T2D management was not objectively ascertained, social desirability bias could have resulted in patient-participants offering the researcher a more positive picture of their situation than is really the case.

## Conclusions

The CCM was largely an effective model to understand patients’ lived experiences of multi-morbidity and incorporating its elements with the ICDM may be explored through more pilot studies. For policymakers, in the short term, integration of chronic services may be through provision of consolidated guidelines for healthcare workers and education material in clinics to improve management of multimorbidity. In the long term, a complete re-organisation of PHC centres may be required where "separate clinics" are phased out and an ideal clinic in which patients with chronic multimorbidities can have a single consultation for all their morbidities in order to decrease the workload associated with chronic multimorbidity and ultimately improve health outcomes.
